# Novel Biomarkers Associated with Deep Venous Thrombosis: A Comprehensive Review

**Published:** 2008-01-21

**Authors:** Dawn M Barnes, Thomas W Wakefield, John E Rectenwald

**Affiliations:** From the Section of Vascular Surgery, Department of Surgery, University of Michigan, Ann Arbor, MI

**Keywords:** deep venous thrombosis, microparticles, D-dimer, P-selectin

## Abstract

Primary and recurrent venous thromboembolic disease (VTE, deep venous thrombosis and pulmonary embolism) remain a significant source of morbidity and mortality in the hospitalized patient. Non-specific subjective complaints and lack of specific objective findings related to acute deep venous thrombosis (DVT) and pulmonary embolism (PE) complicate the diagnosis. There remains no single serum marker available to exclusively confirm the diagnosis of VTE. While D-dimer is highly sensitive and useful for diagnostic exclusion, it lacks the specificity necessary for diagnostic confirmation resulting in the need for a variety of additional studies (i.e.: duplex ultrasound, venography, V/Q scanning, helical thoracic and pelvic CT scans and pulmoary angiography). There is evolving research supporting the utility of various plasma markers as novel “biomarkers” for VTE including selectins, microparticles, interleukin-10 and other cytokines. This review attempts to examine recent literature assessing the utility of P-selectin, microparticles, D-dimer, E-selectin, thrombin, interleukins and fibrin monomers in the diagnosis and guidance of therapy for VTE.

## Introduction

Primary and recurrent venous thromboembolic disease (VTE, deep venous thrombosis and pulmonary embolism) remain a significant source of morbidity and mortality in the hospitalized patient with more than 900,000 primary and recurrent events occurring in 2002 (Table 1). Non-specific subjective complaints and lack of specific objective findings related to acute deep venous thrombosis (DVT) and pulmonary embolism (PE) complicate the diagnosis. There is presently no single serum marker available to exclusively confirm the diagnosis of VTE. The most widely accepted and utilized assay, D-dimer, is highly sensitive and therefore useful for exclusion of the disease but lacks the specificity necessary to confirm the diagnosis. For this reason additional studies including duplex ultrasound, venography, V/Q scanning, helical thoracic and pelvic CT scans and pulmonary angiography remain the standard for diagnosis. Currently, there is evolving research supporting the utility of various plasma markers as novel “biomarkers” for VTE including selectins, microparticles, interleukin-10 and other cytokines. While this review is not presented as a true metaanalysis, a *Pubmed* search was utilized and appropriate studies from 1990 to 2007 were critically reviewed.

## P-Selectin

P-selectin (GMP-140) is an adhesion glycoprotein present in platelet α-granules and endothelial cell Weibel-Palade bodies that is responsible for the initiation of leukocyte rolling. [1] It is up regulated earliest during venous thrombosis and demonstrated in animal models to promote vein wall inflammation. [2] Exposure to an activating stimulus such as thrombin or histamine results in the translocation of P-selectin to the surface of platelets and endothelial cells in a ligand-receptive form. [3, 4] P-selectin glycoprotein ligand 1 (PSGL-1) is a homodimeric mucin found on the majority of leukocytes and is also present in small amounts on platelets. [4,35–36] The binding of P-selectin to PSGL-1 initiates a signal pathway involving tyrosine phosphorylation, MAP Kinase activation and α_m_B_2_ binding activity. This signaling mediates leukocyte-endothelial cell, leukocyte-platelet, leukocyte-leukocyte and, to a lesser degree, platelet-endothelial interactions.

Once *in vivo* transmembrane P-selectin is redistributed onto the surface of activated endothelial cells, it mediates leukocyte rolling and initiates the inflammatory cascade. [7, 19] During thrombosis, the P-selectin expressed on activated platelets present in a thrombus then supports the recruitment of leukocytes. [7, 20] In animal models of thrombosis, P-selectin expression has also been demonstrated to regulate fibrin deposition and thrombus size. [4, 20, 33] Specifically, mice expressing elevated levels of P-selectin have been found to be thrombophilic and P-selectin based therapy has been demonstrated to correct a mouse model of hemophilia A. [4, 7, 34]

In addition to transmembrane P-selectin, a soluble form of P-selectin (sP-sel) may be secreted into the circulation. [5] It may originate from either an alternatively spliced form found in platelets and endothelial cells (lacking a transmembrane domain) [6, 7] or, may be proteolytically cleaved from the cell membrane shortly after activation and therefore may reflect platelet activation. [7–10]. Thus, it has been postulated that plasma levels of sP-sel may predict thrombotic consumptive platelet disorders and also may reflect endothelial cell activation. [7, 11–16] Conversely, other hypotheses speculating on the exact function of sP-sel have included an anti-inflammatory role in which sP-sel directly binds PSGL-1 on leukocytes thereby preventing leukocyte-vessel wall interactions. This sP-sel interaction with activated leukocytes may reduce their ability to adhere to endothelium in vivo. [7, 22–25] In humans, soluble P-selectin appears to be elevated in atherosclerosis and may be predictive of future adverse cardiovascular events. [4, 17–18]

Subramaniam et al. demonstrated that P-selectin deficient mice exhibited a slightly prolonged bleeding time and an increased hemorrhagic response in a local Shwartzman-like reaction suggesting a role for P-selectin in hemostasis. [7, 21] This reaction was induced by injections of bacterial lipopolysaccharide followed by injections of TNF-α into the same skin site one day later and resulted in microthrombi, vascular injury, and hemorrhage at the site of injection. These findings have been supported by a study by André et al. that examined the hemostatic properties of mice genetically engineered to express P-selectin without the cytoplasmic domain (ΔCT mice). [7] In these mice, P-selectin was constitutively expressed on the surface of the endothelial cell and shed from the plasma membrane resulting in a 3- to 4-fold increase of sP-sel in the animals’ plasma. Increased levels of sP-sel appeared to accelerate hemostasis in these mice by activating the coagulation process through the generation of circulating microparticles in plasma. Similarly, it was noted that wild-type (WT) animals infused with a P-selectin-Ig fusion protein (P-sel-Ig) chimera entered a pro-coagulant state as well.

Several studies have demonstrated elevated P-selectin in patients with deep venous thrombosis (DVT). [4, 37–38] Rectenwald et al. measured P-selectin levels in 21 patients with DVT and compared these values to 30 healthy controls and noted them to be elevated at 88.7 ng/mL (compared to 22.1 ng/mL). [4] In a separate study in similar patients, there was a notable decrease in P-selectin (to 54.5 ng/mL) after 7 days of therapeutic heparin therapy. [38]

## Microparticles

Microparticles are small fragments of phospholipids of cell membrane that are shed from platelets, leukocytes, and endothelial cells and are generally less than 1 micron in size that circulate through the plasma of healthy individuals. [4, 26–28] Microparticles are rich in tissue factor (TF), phosphatidylserine and PSGL-1 and thus facilitate and amplify coagulation when recruited into a developing thrombus. [4, 29–31] They also express prothrombinase activity on their membrane [7, 27] and monocyte-derived microparticles have been shown to activate endothelial cells in vitro leading to expression of TF. [7, 32, 33] Elevated levels of circulating microparticles have been associated with several vascular, inflammatory, and coagulation disease processes. Additionally, elevated levels of circulating microparticles are associated with most cardiac risk factors and appear indicative of poor clinical outcome. [66] Platelet derived microparticle levels have generally been considered a marker of platelet activation in cardiovascular disease and studies have demonstrated elevations in microparticles associated with high-risk angiographic coronary obstructive lesions. [65, 67] Certain elevated microparticle phenotypes may also be associated with severity, lesion volume and outcome of acute ischemic stroke. [68]

Finally, elevated levels of microparticles have been recognized in a murine venous thrombosis model. [2, 4] In André et al. study, elevated levels of sP-sel in ΔCT mice enhanced the generation of leukocyte-derived microparticles (Mac-1+), some (13%) of which also expressed TF. Chirinos et al. demonstrated elevated levels of microparticles, elevated microparticle-monocyte conjugates, and increased platelet activation in patients with venous thromboembolism when compared to normal controls. This supports prior studies that suggest released elevated microparticles and their binding to monocytes are key events in thrombogenesis. [69]

## D-Dimer

Numerous published studies and reviews have linked D-dimer, a derivative of cross-linked fibrin, with DVT and PE. In fact, the sensitivity of D-dimer for the diagnosis of venous thromboembolism is reported as 96%–98% (96% for the diagnosis of DVT). [4, 39–41] Khaira and Mann evaluated 80 patients presenting with a clinically diagnosed DVT, 29 of which were diagnosed by venography. [42] Correlating plasma D-dimer levels in this series demonstrated a sensitivity of 96%, a specificity of 40%, a positive predictive value of 48%, and a negative predictive value of 95% when compared to venography. The authors further determined that a normal plasma D-dimer level could be used as a test of exclusion for DVT. Bounameaux et al. applied this concept to aid in the detection of pulmonary embolism in a prospective study involving 171 patients with suspected PE with markedly similar results. [40] In the setting of PE, D-dimer had a 98% sensitivity and 39% specificity and it was reported that a “concentration below 500μg/l rules out the diagnosis of PE”. In 2004, Stein et al. systematically reviewed 78 studies in patients with VTE and correlated D-dimer assays with DVT and PE. [39] The authors compared various D-dimer assays and determined that the ELISA and quantitative rapid ELISA D-dimer assays demonstrated the most clinically useful values. A sensitivity of 96% for DVT and 95% for PE was reported and the authors reiterated that a negative D-dimer assay can safely rule out venous thrombosis with high certainty.

In 2005, Rectenwald et al. hypothesized that plasma microparticles, P-selectin, and D-dimer levels, alone or in combination with patient risk stratification, would accurately predict the presence *or absence* of DVT when compared to the current gold standard of duplex ultrasound examination. [4] A total of 73 patients were enrolled in this pilot study, of which 30 were healthy controls, 22 had acute DVT present on duplex ultrasound and 21 had clinical symptoms supporting DVT but a negative ultrasound. (Tables 2 and 3) The authors established threshold values for all the biomarkers investigated (including D-dimer) that provided the highest sensitivity *while maintaining the highest specificity*: soluble P-selectin values of 0.68 ng/mg per mg total protein, total microparticles levels 125% of control, and D-dimer levels of 3 mg/l. The preliminary data presented suggested that the sensitivity (73%) and specificity (81%) of sP-sel, total microparticles and d-dimer used in combination as dichotomous values for diagnosing DVT, although less sensitive and specific than duplex ultrasound, was an improvement over D-dimer alone (64% sensitivity, 76% specificity).

More recently, D-dimer levels have been investigated for use in guiding the length of therapy for initial venous thromboembolic events (DVT or PE). In 2005, Cosmi et al. investigated D-dimer levels in combination with residual venous obstruction and the risk of recurrence after anticoagulation withdrawal for a first idiopathic DVT. The authors reported that the optimal duration of oral anticoagulation therapy after a first episode of venous thromboembolism (VTE) is still a matter of debate and while extending oral anticoagulation therapy reduces the risk of recurrence by 90%, it is associated with an increased clinically important risk of major bleeding. [43, 44] Specifically, this prospective study examined 400 patients that completed a ‘therapeutic course’ of oral anticoagulant therapy for a first episode of idiopathic proximal DVT and had evidence of residual venous obstruction by ultrasound on the day of oral anticoagulant suspension. D-dimer levels were followed at 30 days and the overall recurrence rate was 16.7%. The authors demonstrated that abnormal D-dimer levels at one month after therapy withdrawal are an independent risk factor for recurrent VTE (multivariate hazard ratio, HR, of 3.32). They also noted that residual venous obstruction on duplex ultrasound at the time of oral anticoagulation withdrawal, despite a normal or abnormal D-dimer after one month, does not influence the rate of recurrence.

This observation was followed in 2006 by the PROLONG study that utilized D-dimer testing to determine the duration of anticoagulation therapy. [45] 608 patients with a first episode of symptomatic, unprovoked venous thromboembolism were examined after completing at least 3 months of therapy with warfarin and were followed for a mean duration of 1.4 years. The 385 patients with normal D-dimer levels at the conclusion of therapy did not continue with any further anticoagulant treatment, and 4.4% of these patients demonstrated a recurrent venous thrombotic event. The 223 patients with abnormal D-dimer levels were randomized to two groups: 103 resumed anticoagulation and 120 discontinued therapy. 2.9% of those patients that continued anticoagulation suffered a recurrent thromboembolic event compared to a 15% recurrence in the non-anticoagulated group. In this series, event rates were significantly higher among patients with abnormal D-dimer levels that stopped anticoagulation than among those who continued anticoagulation (adjusted hazard ratio 4.26) or among those with normal D-dimer levels (adjusted HR 2.27) and the authors support that there is a clear benefit of prolonged therapy with Vitamin K antagonists in patients whose D-dimer levels are abnormal one month after the discontinuation of this treatment.

## E-Selectin

E-selectin temporally follows P-selectin up-regulation and has been noted to augment the thrombotic response in a murine model of venous thrombosis and to amplify the effects of P-selectin. [2,46] In 2003, Myers et al. examined 659 male mice that underwent IVC ligation to induce thrombosis. For this experiment, mice were separated into 4 groups: wild type controls (WT), mice with elevated circulating levels of soluble P-selectin (Δ-CT), P-selectin gene-interrupted knockout mice (PKO) and E- and P-selectin gene-interrupted mice (EPKO). As previously noted, the Δ-CT mice generated 50% more thrombus than wild type mice. All mice demonstrated a doubly-labeled leukocyte (MAC-1) and platelet (CD41)-derived microparticle (MP) population with the exception of the EPKO mice which produced a primarily platelet-derived, and a smaller leukocyte-derived, MP population with an associated significant decrease in thrombus mass.

Jilma et al. more recently has demonstrated that homozygosity of the single nucleotide polymorphism *Ser*128*Arg* in the E-selectin gene in humans may be associated with recurrent venous thromboembolism. [47] This polymorphism alters ligand affinity, enhances myeloid cellular tethering, and regulates leukocyte-endothelial cell interactions *in vitro*. [47–50] E-selectin polymorphism has been associated with enhanced endotoxin-triggered, tissue factor-mediated coagulation in humans, atherosclerosis, myocardial infarction and restenosis after angioplasty and may be associated with recurrent VTE. [47, 50–55] In this study, 585 patients with a first idiopathic VTE were prospectively examined of which 102 (17%) were heterozygous for the Ser128Arg mutation and 11 (2%) were homozygous. Of the total patient population, 90 patients (15% of 585 patients) demonstrated a recurrent VTE. Homozygosity for this mutation appeared to increase the cumulative likelihood for early recurrent VTE and was considered an independent predictor or recurrent VTE (HR 4.1) compared to a HR of 1.1 for the heterozygous population.

## Thrombin

Generation of thrombin is a well established pivotal step of hemostasis. It is also clear that there are various risk factors for thrombin generation that are associated with recurrent VTE. [56] Because of the observation that reduced thrombin generation has been recorded in patients with bleeding tendencies and increased thrombin generation has been noted in patients at risk for VTE, Hron et al. hypothesized that by measuring thrombin generation, patients with VTE could be stratified into high- and low-risk categories for recurrence. The authors prospectively examined 914 patients with a first, spontaneous VTE and assessed the relationship between thrombin generation and risk of recurrence. They found that 100 patients (11%) suffered recurrent VTE and that patients without recurrent VTE had lower thrombin generation (as measured by a commercially available thrombin assay system) than those with recurrence (mean 349.2 nM compared to 419.5 nM, p = <0.001). Moreover, when compared to patients with thrombin generation >400 nM, those with values between 300–400 nM had a relative risk of recurrence of DVT of 0.42 and this further decreased to 0.37 for those patients with values <300 nM. Even after four years, the probability of recurrence was still reduced at 6.5% among patients with thrombin generation <400 nM (2/3 of patients) compared to 20% for values >400 nM. This suggests that patients with values <400 nM probably do not benefit from indefinite therapy with vitamin K antagonists.

## Interleukin-10

Interleukin-10 (IL-10) is a major immunoregulatory and anti-inflammatory cytokine with a highly polymorphic promoter gene with two polymorphic dinucleotide repeats (IL-10G and IL-10R microsattelites). [59–63] It has been suggested that these polymorphisms may play a role in the onset of various autoimmune and lymphoproliferative disorders. [59] Animal models have demonstrated that IL-10 modulates the development of DVT. [59, 64] Cochery-Nouvellon et al. recently conducted a human case-control study to evaluate whether IL-10 polymorphisms is a risk factor for venous thrombosis as this pathology is certainly associated with a consistent inflammatory state. They compared 74 patients with at least one episode of venous thrombosis to 100 healthy controls and conducted multivariate logistic regression analysis. The authors demonstrated that IL-10 G13 and G10 alleles are independent risk factors for venous thrombosis (Odds ratio 3.33 and 2.83 respectively). Furthermore, they found that the IL-10 G10 allele was more frequent in recurrent disease suggesting that it may be an independent risk factor for recurrent disease.

## Fibrin Monomers

Vogel and Spanuth assessed fibrin monomers as markers of intravascular activation of the coagulation system resulting in disseminated intravascular coagulation or deep venous thrombosis. [70] Specifically, elevated levels of fibrin monomers following orthopedic surgery were determined to have a 63–73% positive predictive value for the diagnosis of post-operative DVT, possibly allowing for early detection of a prethrombotic state and preevent prophylaxis. While fibrin monomers more recently are felt to be inferior to D-dimer assays as a primary screening tool for DVT, they may aid in this diagnosis when combined with both ultrasound and D-dimer assays. [71]

## Conclusion

Historically, the diagnosis of acute venous thromboembolism (DVT and PE) has relied on primarily imaging modalities to establish the diagnosis of VTE. A multi-modality approach, involving not only imaging but also serology, is currently evolving. There is mounting evidence to support the use of certain serum “biomarkers” (i.e. selectins, microparticles, thrombin, D-dimer, and IL-10) that may aid in this diagnosis and treatment of DVT and PE. Regardless of the difficulties in diagnosis of VTE, issues surrounding the management of patients with VTE and the duration of treatment remain problematic. Currently, a third of patients will experience recurrent VTE within the next 5 to 8 years with an associated 5% case-fatality rate of recurrence of VTE in patients that have completed standard treatment. [56–58] Recent studies support that these same biomarkers may not only aid in diagnosis, but may in the future help guide venous thromboembolic therapy by identifying patients at high risk of recurrence that may benefit from prolonged or indefinite therapy with anticoagulation. In next few years, biomarkers may help predict which thrombi will resolve spontaneously or recanalize, and those that will not—potentially identifying patients that would benefit from more aggressive therapies than standard anticoagulation.

## Figures and Tables

**Table 1.** Total US 2002 VTE events—incident & recurrent, fatal & non-fatal.

**Event**	**Community-acquired**	**Hospital-acquired**	**Total**
Non-fatal VTE	193,598	419,825	613,423
DVT	108,240	268,125	376,365
PE	85,358	151,700	237,058
Fatal VTE	106,551	189,819	296,370
DVT	649	1609	2258
PE	105,902	188,210	294,112
Grand Total	300,149	609,644	909,793

Unpublished 2002 data from the University of Michigan describing the incidence of VTE (venous thromboembolic events) including both initial and recurrent episodes; DVT = deep venous thrombosis; PE = pulmonary embolism; events occurring with 90 days after hospitalization were categorized as hospital-acquired. Heit JA, Cohen AT, Anderson FA Jr. Blood 2005; 106(11):abstract #910, p. 267a.

**Table 2.** Use of P-selectin, total microparticles and D-dimer as dichotomous variables for the prediction of DVT.

**Variables**	**Threshold value**	**Sensitivity**	**Specificity**	**Accuracy**
Soluble P-selectin	0.68 ng/mg TP	68%	81%	74%
Total microparticles	125% (compared to controls)	50%	67%	58%
D-dimer	3 mg/l	64%	76%	70%
Combined variables		73%	81%	77%

Logistic regression evaluating DVT and symptomatic patients using dichotomous variables establishing threshold values for data analysis. Adapted from Rectenwald JE, Myers DD, Hawley AE et al. D-Dimer, P-Selectin, and microparticles: Novel markers to predict deep venous thrombosis. Thromb Haemost 2005; 94:1312–17.

**Table 3.** Use of P-selectin, total microparticles and D-dimer as continuous variables for the prediction of DVT.

**Variables**	**Sensitivity**	**Specificity**	**Accuracy**
Soluble P-selectin	71%	81%	76%
Total microparticles	59%	62%	61%
D-dimer	54%	81%	67%
Combined variables	81%	62%	71%
Combined variables+ risk score	62%	57%	60%

Logistic regression evaluating DVT and symptomatic patients using continuous variables. Adapted from table 4, Rectenwald JE, Myers DD, Hawley AE et al. D-Dimer, P-Selectin, and microparticles: Novel markers to predict deep venous thrombosis. Thromb Haemost 2005; 94:1312–17.
